# Carbon dynamics under loss and restoration scenarios in the world’s largest seagrass meadow

**DOI:** 10.1038/s41598-025-01993-1

**Published:** 2025-05-16

**Authors:** Monica M. Moritsch, Austin J. Gallagher, S. David Harris, Wells Howe, Chuancheng Fu, Tadzio Bervoets, Carlos M. Duarte

**Affiliations:** 1 Beneath The Waves, 3 Austin St., PO Box 290036, Boston, MA 02129 USA; 2 Marine Science Program, Biological and Environmental Science and Engineering Division (BESE), King Abdullah University of Science and Technology (KAUST), Thuwal, Kingdom of Saudi Arabia; 3 Caribbean Biodiversity Fund, Nassau, The Bahamas

**Keywords:** Blue carbon, InVEST, Seagrass, Climate change, Carbon accumulation, Natural climate solutions, Carbon cycle, Ecological modelling, Marine chemistry, Ocean sciences, Marine biology, Marine chemistry

## Abstract

Seagrass sediments accumulate high amounts of organic carbon, but they are threatened by human activities and their global extent continues to shrink. Simultaneously, there is interest in including seagrass carbon accumulation in countries’ Nationally Determined Contributions (NDCs). We used the InVEST Coastal Blue Carbon Model to estimate sediment organic carbon (SOC) accumulation over 100 years in seagrass of the Bahama Banks, the world’s largest seagrass meadow. Using seagrass maps and sediment core measurements, we modeled SOC accumulation in two scenarios: (1) 1% seagrass area loss per year, the Business As Usual scenario (BAU); (2) restoration of seagrass extent to that of 30 years prior by 2120, meeting the goals of the Kunming-Montreal global biodiversity framework. With a conservative initial seagrass extent, by 2120, the SOC accumulation was 90.6 Mt CO_2_ eq (24.0 autochthonous Mt CO_2_ eq) in the BAU and 703.7 Mt CO_2_ eq (186.5 autochthonous Mt CO_2_ eq) in the restoration scenario, and average additional SOC accumulation was 611.0 Mt CO_2_ eq (161.9 autochthonous Mt CO_2_ eq). Using a high estimate of initial seagrass extent, by 2120, the net SOC accumulation was 155.4 Mt CO_2_ eq (41.2 autochthonous Mt CO_2_ eq) in the BAU and 1058.2 Mt CO_2_ eq (280.4 autochthonous Mt CO_2_ eq) in the restoration scenario, and additional SOC accumulation was 902.8 Mt CO_2_ eq (239.2 autochthonous Mt CO_2_ eq). The potential for either SOC accumulation or losses to occur if seagrass extent continues to decline highlights uncertainty around whether Bahamian seagrass meadows will remain a net carbon sink. The additional accumulation of autochthonous carbon if seagrasses were restored was comparable in scale to the annual greenhouse gas emissions of The Bahamas, suggesting potential for seagrass restoration to contribute to the country’s NDCs and broader climate mitigation strategies.

## Introduction

Decarbonization of human activities at a global scale is necessary but insufficient to keep warming below 2^o^C, requiring efforts to meet climate goals to mitigate emissions as well as activate carbon dioxide removal from the atmosphere^1^. There is a large potential role for natural climate solutions (NCS) to contribute to meeting this goal, which encompasses ecosystem management actions to reduce greenhouse gas (GHGs) emissions or sequester carbon dioxide (CO_2_) from the atmosphere. These actions can take the form of restoration, conservation, or shifts in management practices to promote GHG mitigation^2^. Blue carbon ecosystems (BCEs), or vegetated coastal and marine ecosystems such as salt marshes, mangroves, and seagrasses, offer the ecosystem service of GHG drawdown through accumulation and preservation of organic carbon in their sediments.

While there is wide global variation in sediment organic carbon (SOC) per hectare^3^, some seagrass meadows can store over 300 t CO_2 _eq ha^−1^, similar to terrestrial forests^4,5^ and accumulate over 5 CO_2_ eq ha^−1^ yr^−1^^6^, 1–2 orders of magnitude higher than terrestrial forests (0.003–0.048 Mg CO_2 _eq ha^−1^ yr^−1^)^7^, making them an efficient NCS despite their comparatively small area if managed to promote long-term carbon storage. Their dense network of aboveground biomass and belowground roots captures sediment and detritus, leading to regular accumulation of SOC, while the water-logged environment slows the decomposition that produces GHG emissions and leads to SOC storage on the scale of millennia^3,7^. However, when sediments are disturbed and exposed to oxic environments, emissions associated with decomposition can occur at a much higher rate^8^. SOC storage and accumulation rates are influenced by species, hydrologic conditions, and geomorphic setting^4^. SOC accumulation can occur autochthonously through primary production in seagrass. Additionally, a large portion of seagrass meadows’ SOC accumulation can be allochthonous material, which originates from other marine or terrestrial ecosystems and is subsequently transported to the seagrass meadow^4,9^. While a portion of allochthonous SOC is likely not reactive, seagrass meadows may also preserve some allochthonous organic matter that would otherwise decompose and return to the atmosphere as GHGs^4,9^. In seagrass systems, autochthonous carbon generally ranges from 38 to 67%, but as little as 5% has been observed, with correspondingly large proportions of allochthonous carbon^4,9,10^. For example, in Bahia San Quintín in northern Mexico, > 80% of OC in sediments of lagoonal seagrass beds derived from marine phytoplankton, while contributions from seagrass itself were minimal^10^.

The climate mitigation capacity of BCEs can be included in Nationally Determined Contributions (NDCs) for meeting commitments toward the Paris Climate Agreement^11,12^. Whether all SOC or only autochthonous SOC can be included in NDCs and other carbon accounting frameworks is specific to the context of sediment deposition (and to jurisdictional rules). To qualify as “additional” organic carbon accumulation, a management action must either prevent emissions of GHGs or must generate drawdowns of atmospheric CO_2_. For allochthonous carbon in BCEs to satisfy the condition of additionality, the BCE must capture and store organic carbon that would remain reactive and return to the atmosphere if the BCE were not present, which may be challenging to demonstrate due to logistical and cost constraints^13–15^. In seagrass, this is accomplished when carbon-rich material from other ecosystems, including tidal marshes and mangroves, is deposited due to the dense network of biomass and roots and then preserved in anoxic sediments^10,14,16^. The fraction of allochthonous carbon that would degrade without burial is highly dependent on the biogeochemical properties of the organismal tissue and environmental conditions. While the recognition of only autochthonous SOC in accounting frameworks presents a conservative calculation of BCE’s climate mitigation, the exclusion of allochthonous SOC can underestimate the climate benefits of seagrass^10,13^.

Seagrass is mentioned as part of the climate mitigation actions for at least five countries’ NDCs, including The Bahamas^17^. In 2022, the Parliament of The Bahamas passed three pieces of legislation to establish a carbon trading market as a mechanism for meeting its NDCs. This bill allows the ability to generate carbon and natural asset credits in coastal and marine ecosystems^18^. Since the recent discovery of extensive seagrass meadows within the country’s waters, there is growing interest from government agencies and conservationists to ascertain the amount of carbon accumulation and storage that Bahamian seagrass could provide and how it may be leveraged for NDCs.

Since the start of the 20 th century, 19 to 29% of the world’s seagrass area has been lost^19,20^. Global loss rates accelerated in the 1990’s and early 2000’s from 1.5 to 7% per year^20^. Losing the top 1 m of seagrass sediments to erosion at this accelerated rate could result in estimated emissions of up to 300 Mt CO_2_ eq yr^−1^ if all of the SOC were remineralized and returned to the atmosphere as CO_2_^3^, potentially exceeding the total annual accumulation by BCEs^7^. Seagrass is vulnerable to sediment loading in the water column and nutrient pollution, both of which contribute to seagrass declines globally^17,21,22^. Coastal development and recreational boating create additional stress by increasing pollution levels or mechanically damaging meadow structure with boat propellers^17,23^. Protection alone will not result in major regrowth of seagrass; restoration is necessary to achieve substantial recovery^24^, with the Kunming-Montreal global biodiversity framework targeting protection for 30% of exiting ecosystems and restoration of 30% of degraded ecosystems by 2030. To meet targets, plans must be made for restoration by 2030, even if the restoration process will take decades^25^.

The Bahamas possesses the world’s largest seagrass meadow, which spans up to 92,500 km^2^, offering large potential for SOC accumulation^16,26^. The tropical Atlantic region loses an estimated 1% of its seagrass area annually^20^. While it is difficult to estimate historical seagrass area for the country’s waters, seagrass decline in The Bahamas has been documented through direct measurement of area^27,28^ and inferred through decline of seagrass carbon contributions to sediments^16^. In the last 30 years, anthropogenic divers correlated with loss have included deteriorated water quality from nutrient pollution and physical damage associated with tourism, development, and vessel activities^28,29^. The number of annual visitors to The Bahamas grew by 20% from 2003 to 2019, though it declined sharply during the Covid-19 pandemic^30^. Development, dredging, shipping, and fishing activity to support the growing tourism industry have led to disturbance of bottom sediments, which may promote erosion of existing SOC stocks, along with potential nutrient pollution that contributes to seagrass health declines through light reduction and algal smothering^23,31,32^.

**Fig. 1:**
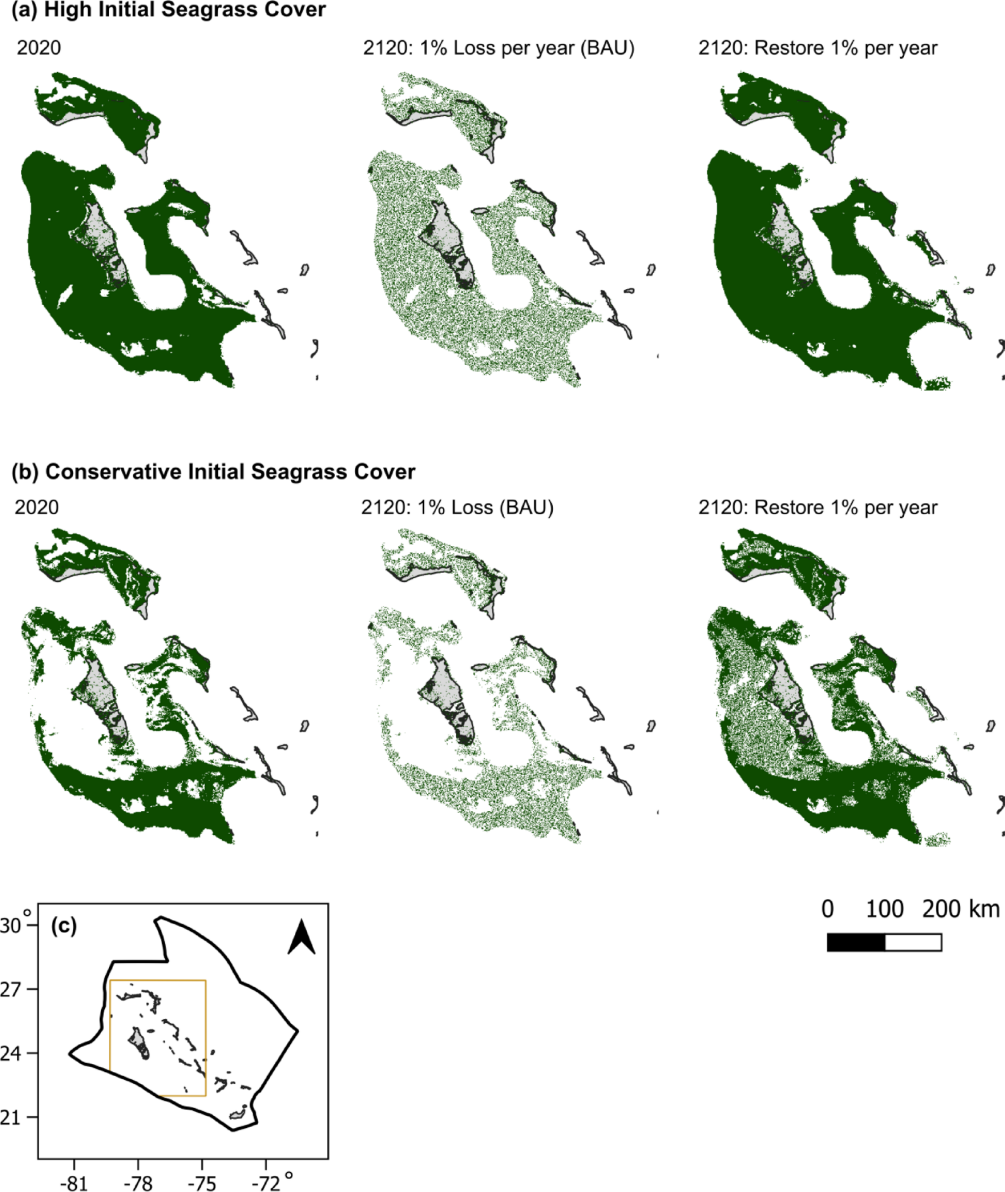
Seagrass extent in the Bahama Banks for **(a)** high and **(b)** conservative estimates (Gallagher et al. 2022). Seagrass is represented with dark green shading. Scale is represented in the bar on the lower right. **(c)** Area map of the Exclusive Economic Zone (EEZ) of The Bahamas (thick black outline) and modeled area (thin brown outline). Maps were created in QGIS 3.36.0 (www.qgis.org).

Future projections of SOC accumulation and stocks are critical to establishing baselines against which to measure additional SOC accumulation and the additionality of management actions. While current accumulation rates and stocks have been measured in the Bahama Banks, there are, as of yet, no projections of future SOC accumulation (and its subsequent contribution to global climate and biodiversity protection goals) that may occur with further losses or restoration action. Here we developed estimates of future SOC accumulation in Bahamian seagrass ecosystems (Fig. 1) using a spatially explicit modeling framework. We aimed to calculate additionality, or relative differences in SOC accumulation relative to a “Business As Usual” (BAU) scenario of prevailing trends in seagrass decline, for a restoration scenario that would meet the targets of the Kunming-Montreal framework. This scenario would conserve all existing seagrass and reverse the seagrass degradation of the prior 30 years based on the prevailing 1% loss rate, restoring an equivalent of 34.8% of the current seagrass extent. We present both autochthonous SOC and total SOC accumulation to quantify seagrass climate mitigation under the current paradigm of carbon accounting frameworks and under a broader view of carbon accumulation in BCEs.

## Results

### Sediment organic carbon accumulation

**Fig. 2:**
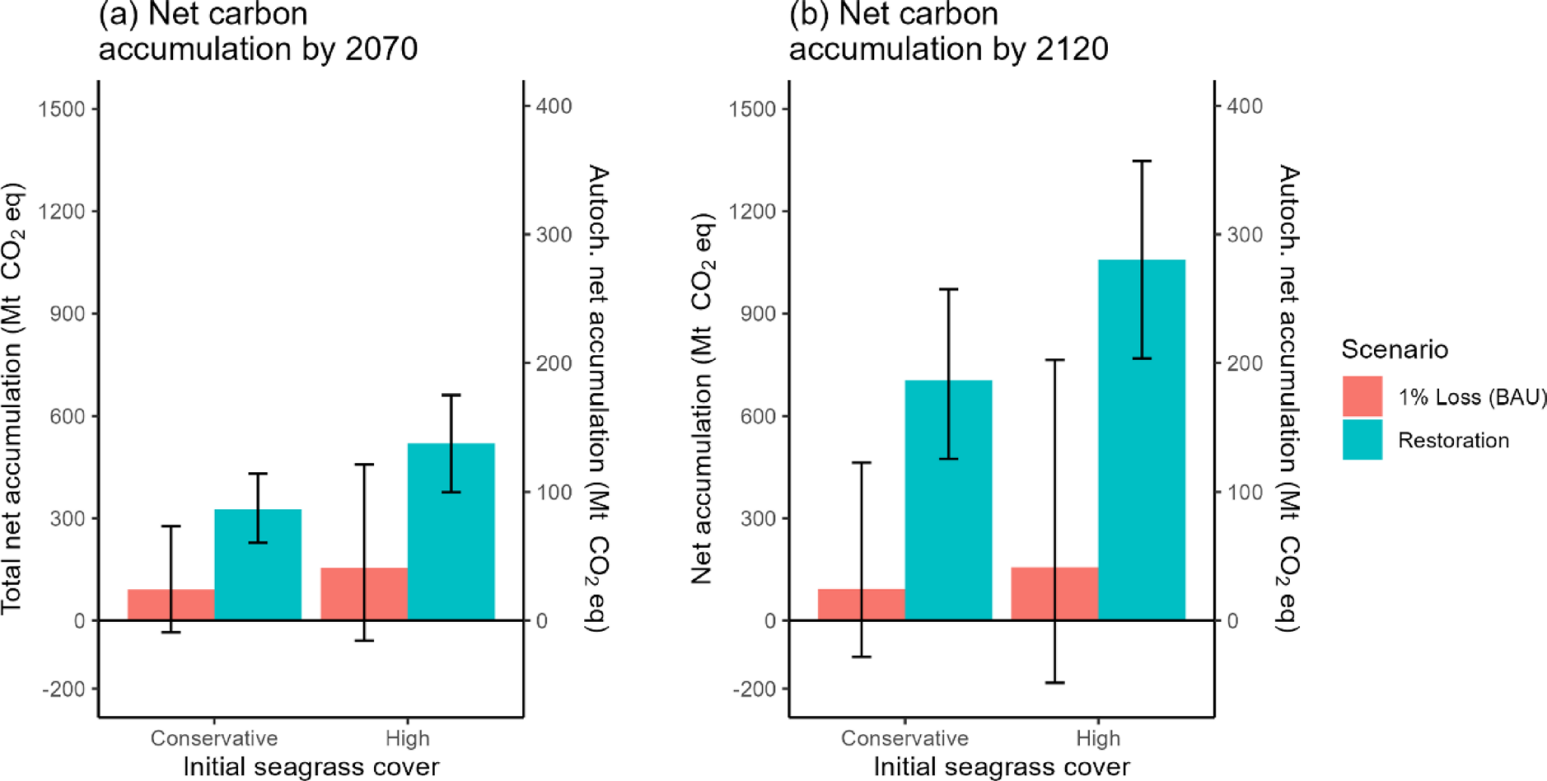
Total net SOC accumulation for the four scenarios and two initial seagrass cover estimates by **(a)** 2070 and **(b)** 2120. Negative numbers represent net loss of SOC. Bars represent upper and lower bounds based on ± 0.5% annual rates of change, ± 1 standard error (SE) sediment organic carbon stock, and ± 1 SE carbon accumulation rate.

**Fig. 3:**
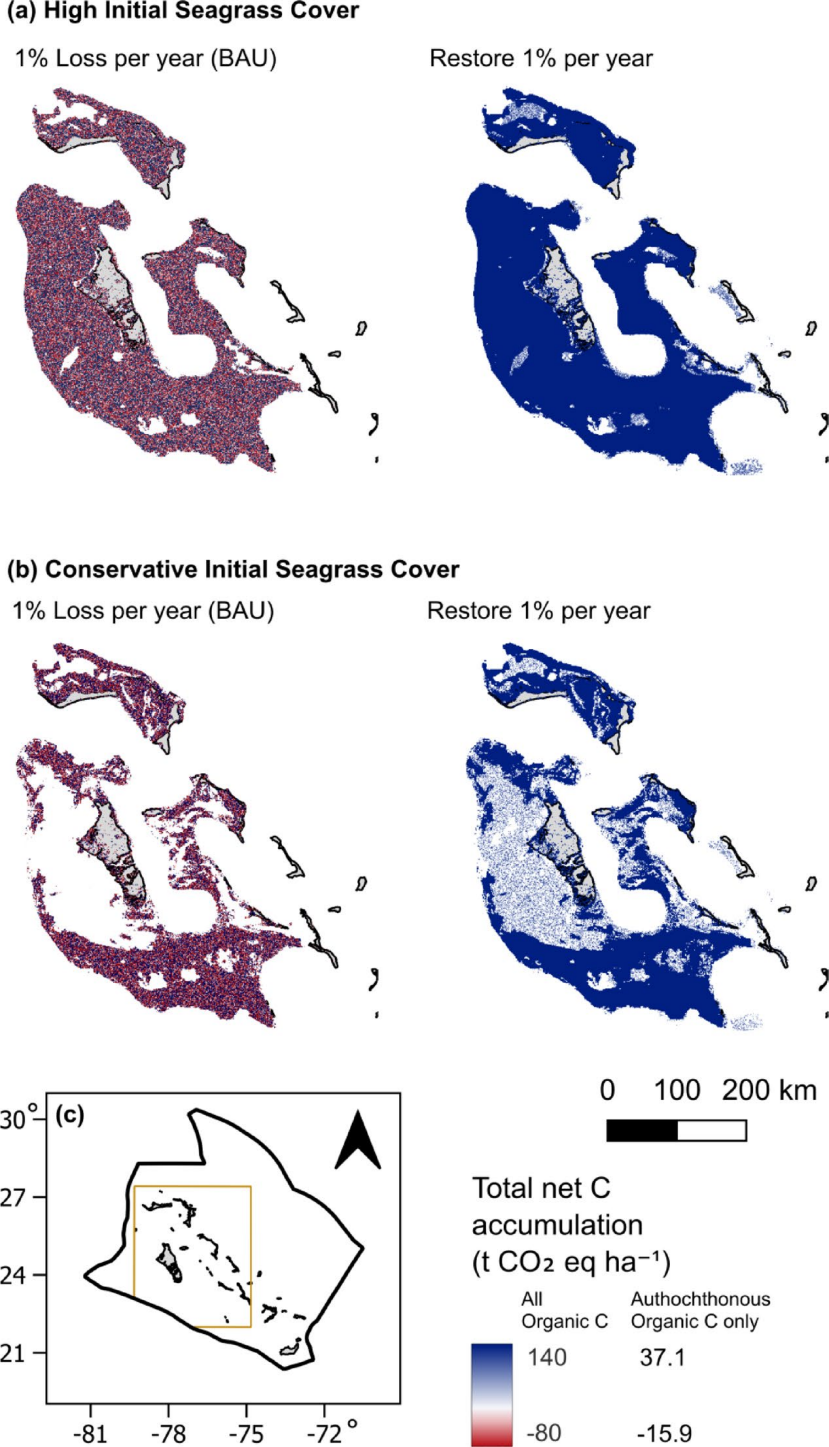
Seagrass SOC accumulation maps for **(a)** high and **(b)** conservative estimates. Blue areas represent net SOC gain and red areas represent net SOC loss. Scale is represented in the bar on the lower right. (c) Area map of the Exclusive Economic Zone (EEZ) of The Bahamas (thick black outline) and modeled area (thin brown outline). BAU: Business As Usual. Maps were created in QGIS 3.36.0 (www.qgis.org).

On average, positive net SOC accumulation occurred for both scenarios in 2070 and 2120 (Fig. 2). Despite the seagrass area continuing to decline in the BAU scenario, the average SOC accumulation by the remaining seagrass exceeded SOC losses from disturbance. However, the lower SOC estimates for the BAU were negative, indicating net SOC loss when seagrass declined at 1.5% annually. For conservative and high initial seagrass extent, average total SOC accumulation increased slightly over time from 90.6 (−34.5 to 276.9) and 153.8 (−59.0 to 458.0) Mt CO_2_ eq in 2070 (Fig. 2a) to 92.7 (−106.6 to 463.0) and 155.4 (−182.9 to 763.5) Mt CO_2_ eq in 2120 (Fig. 2b), respectively. However, for the lower estimate, SOC accumulation declined over time. Autochthonous SOC correspondingly increased from 24.0 (8.7 to 39.3) Mt CO_2_ eq and 40.8 (15.1 to 66.4) Mt CO_2_ eq in 2070 to 24.6 (2.0 to 47.2) Mt CO_2_ eq and 41.2 (3.5 to 78.9) Mt CO_2_ eq in 2120 (Fig. 2). Loss of seagrass cover early in the century led to losses of up to 79.3 total t CO_2_ eq ha^−1^, or 21.0 autochthonous t CO_2_ eq ha^−1^, while seagrass that remained for the full 100 years accumulated up to 140.0 total t CO_2_ eq ha^−1^, or 37.1 autochthonous t CO_2_ eq ha^−1^ (Fig. 3).

There was substantial overlap in the ranges of SOC accumulation for conservative and high seagrass extents within the same scenario, but accumulation diverged between scenarios by 2120. The 2120 upper bound of accumulation in the BAU scenario was slightly below the lower bound of accumulation in the restoration scenario (Fig. 2b).

The restoration scenario accumulated more SOC than the BAU scenario by 3.4 to 3.6 times in 2070 and 6.8 to 7.6 times in 2120 (Fig. 2). The net SOC accumulation for conservative and high initial seagrass extent was 325.6 (228.7 to 431.1) and 518.7 (376.6 to 660.5) Mt CO_2_ eq in 2070 (Fig. 2a) and 703.7 (474.1 to 971.1) and 1058.2 (768.1 to 1347.5) Mt CO_2_ eq in 2120 (Fig. 2b), respectively. Seagrass that was restored early in the modeling period accumulated up to 124.3 t CO_2_ eq ha^−1^ (32.9 autochthonous t CO_2_ eq ha^−1^), or 89% of the SOC accumulation of undisturbed seagrass by 2120 (Fig. 3).

**Fig. 4:**
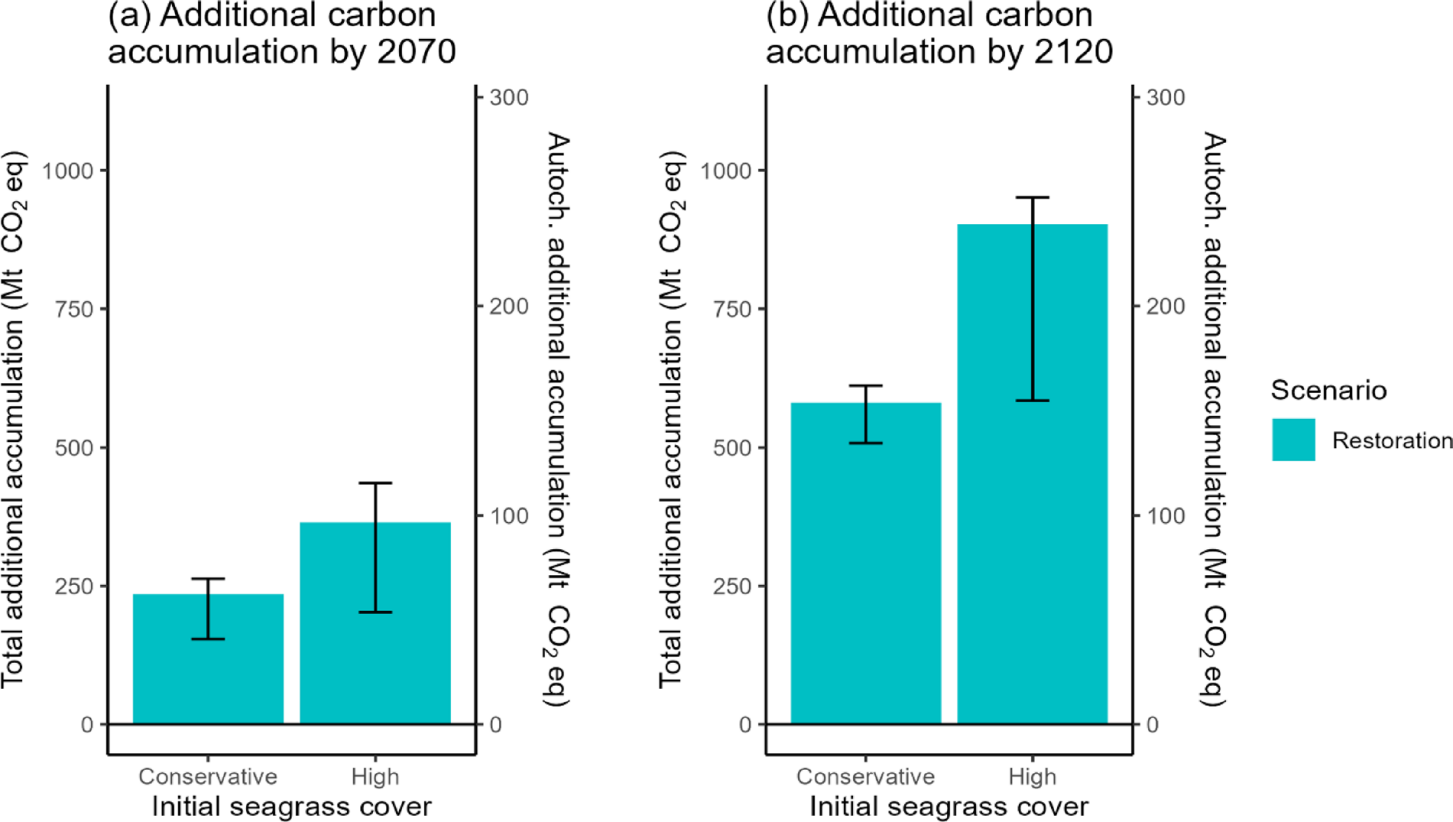
Differences in net SOC accumulation of seagrass sediments for two initial seagrass cover estimates relative to the Business As Usual scenario of 1% loss per year by **(a)** 2070 and **(b)** 2120. Bars represent upper and lower bounds based on ± 0.5% annual rates of change, ± 1 standard error (SE) sediment organic carbon stock, and ± 1 SE carbon accumulation rate.

Total additional SOC accumulation ranged from 508.0 to 951.8 Mt CO_2_ eq by the end of 100 years, and autochthonous additional SOC ranged from 134.6 to 252.0 Mt CO_2_ eq at the same time point. Conservative and high estimates of additional SOC were somewhat similar in 2070, but by 2120, they had diverged (Fig. 4).

**Fig. 5:**
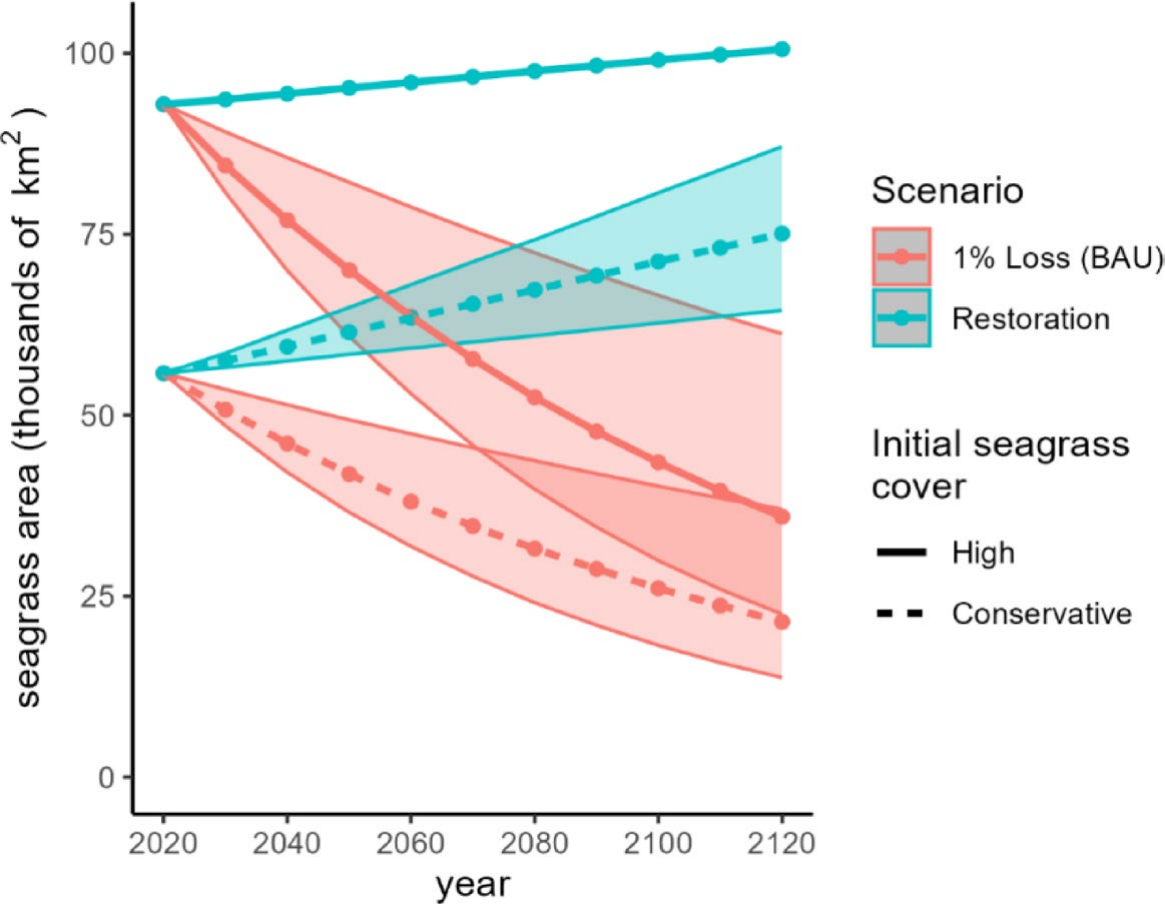
Projected seagrass area over time for each scenario and initial seagrass cover estimate. Shaded area represents 1.0 ± 0.5% annual rate of change. In the restoration scenario with high initial cover, rates 0.5 to 1.5% annual gain exceeded the maximum available area for restoration; restoration progresses until the maximum area is reached.

Timing of seagrass loss was a major driver of SOC accumulation. Keeping seagrass for longer allowed more SOC accumulation before losing SOC to disturbance, whereas early disturbance of large quantities of seagrass reduced the likelihood that the remaining seagrass could compensate for lost SOC. In the BAU scenario, declines in seagrass area began to slow around 2080, with the conservative and high seagrass estimates approaching similar extents (Fig. 5). By 2120, the BAU scenario lost 34,300 to 57,100 km^2^ of seagrass.

In the restoration scenario, seagrass area increased by 19,300 km^2^ using the conservative initial seagrass estimate and by 7,600 km^2^ using the high initial estimate, resulting in a total of 75,000 to 101,000 km^2^ by 2120 (Figs. 1 and 5). Seagrass area gains were greater using the conservative initial estimate for seagrass cover because more seagrass-free area at suitable depths was available to be restored, though total hectares it did not surpass the those of the high initial seagrass area at any time.

## Discussion

The range of SOC accumulation in the BAU scenario spans positive and negative values. Therefore, it is uncertain whether the Bahama Banks seagrass will be a carbon sink or become a net source of emissions if losses in area continue for 100 years, particularly if annual rates of decline accelerate to 1.5% or more. Conversely, if rates slow to 0.5% or restoration occurs to reverse the losses, seagrass has the potential to remain a net sink for SOC (Fig. 2). SOC accumulation early in the century by remaining seagrass helped to slow net SOC losses over time, indicating that conservation efforts to avoid or delay seagrass loss as long as possible can reduce net emissions over the next century. Likewise, the timing of seagrass restoration influenced the total climate mitigation. Seagrass that was restored early in the century was able to accumulate nearly 90% of the SOC of undisturbed seagrass. This suggests that while restored seagrass takes years to decades to recover to its former SOC accumulation abilities^33^, seagrass restoration that meets the targets of the Kunming-Montreal framework can still play a substantial role in recovering or augmenting the ecosystem service of climate mitigation.

### Sources of uncertainty

The analysis was sensitive to the carbon accumulation rates (CARs) and rates of seagrass gain and loss used. We noted a lack of published sediment cores from *Halophila spp.* and *Halodule spp.* within the Bahama Banks, highlighting a data gap for how CARs for different species compare and how vegetation density influences CARs. We assumed that loss and restoration rates would continue at the same pace (until the maximum restorable area was reached). We also assumed that CARs would be constant for 100 years. However, SOC accumulation can be punctuated by intermittent erosion events such as coastal storms, the severity and frequency of which are expected to increase under climate change^34^.

Neighboring ecosystems such as mangroves can contribute carbon to seagrass sediments or mitigate erosional forces, contributing to variation in SOC accumulation and loss rates within seagrass beds at local scales^16,35^. Our estimates of SOC losses were also sensitive to SOC remineralization rates when sediments are disturbed. Remineralization of OC in eroded sediment is difficult to track once it is transported away from BCEs, and its quantification has been largely dependent on models. A fraction may be redeposited in similarly anoxic environments or transported to the deep sea, where it will not interact with the atmosphere for a century or more. This fraction is highly dependent on local geomorphic features, the surrounding microbial community, and water transport patterns. Furthermore, the molecular composition of the SOC, presence of any mineral associations, and particle size influence its resistance to degradation^8,36,37^. However, export of SOC does not necessarily equate to immediate emissions. Resuspended sediment may resettle in another location prior to oxidation or be advected into the deep ocean, where it can stay for climate-relevant timescales^38,39^. The probability of carbon advection to the deep ocean is higher when the location of sediment resuspension is close to waters > 1000 m deep^40^, as is the case at the eastern edges of the Bahama Banks.

In the Caribbean Sea and Atlantic Ocean, warming waters are decreasing the difference between average ambient temperatures and seagrass’ thermal limits^41^. Warmer waters may also increase the prevalence of wasting diseases that negatively impact biomass and photosynthetic surface area^42^. In contrast, ocean acidification may increase CARs. Seagrass exposed to lower pH levels can increase shoot density and both aboveground and belowground biomass^43^. Next, the grazer community plays a large role in regulating biomass and soil bulk density in BCEs^44–46^. Grazer populations face their own pressures from changing conditions^47,48^. Next, the predator community may play a role in regulating Bahamian seagrass sediment SOC stocks. Locations where tiger sharks spend more time have higher SOC stocks than areas where they are found less frequently, though the mechanisms behind this pattern are unknown^49^. Nutrient enrichment is another key pressure on seagrasses. Changes in the magnitude of terrestrial runoff could deliver greater nutrients to seagrass beds and promote algal overgrowth^22^. Thus, overall effects of climate change on the magnitude of SOC accumulation will likely depend on an interplay of seagrass extent, temperature, local water chemistry, and community composition.

Unlike what we have modeled here, spatial patterns of seagrass loss and restoration are not random. Declines in seagrass cover are often non-linearly associated with the intensity of environmental and anthropogenic stressors and can exhibit sudden “tipping point” changes^50,51^. Locations for restoration are also not random, determined by many factors including physical conditions, site accessibility, bathymetry, predator-prey relationships, funding, jurisdiction, and social preferences^52,53^. Further research is needed to clarify the spatial patterns of CARs and potential SOC losses in The Bahamas. The Intergovernmental Panel on Climate Change (IPCC) suggests that protocols for calculation national GHG inventories incorporate some degree of local specificity (i.e., Tier 3 methodologies). Approaches of intermediate complexity that incorporate country-specific data (i.e., Tier 2 methodologies) are allowed when local data are limited or logistically difficult to obtain^54,55^. Our estimates represent Tier 2 complexity, as we assumed CARs for mature seagrass were uniform across the Bahama Banks based on sediment cores taken from within the study area.

### Implications for climate policy in small Island developing States

If seagrasses are restored to their former extent of 30 years ago, before a decline in carbon burial rates was observed^16^, the additional SOC accumulation could serve to counter national GHG emissions, assuming that the legal conditions for offsetting are satisfied. Our high estimate for annual autochthonous seagrass carbon additionality in the restoration scenario, 2.4 Mt CO_2_ eq yr^−1^ (Fig. 4b), was comparable to The Bahamas’ average annual emissions from 2016 to 2020: 2.7 Mt CO_2_ eq yr^−1^^56^. If counting SOC accumulation from all sources, our upper estimate of 9.5 Mt CO_2_ eq yr^−1^ additional carbon (Fig. 4b) is more than triple the magnitude of national emissions. Both estimates suggest that while restoration can take multiple decades to reach full SOC accumulation potential, seagrass restoration could aid the country in its pursuit of carbon neutrality over the next 100 years. Meanwhile, continuation of seagrass losses could undermine the ability of seagrass to contribute to climate mitigation and The Bahamas’ NDCs by releasing stored carbon.

The costs of seagrass restoration can be prohibitive for Small Island Developing States (SIDS) and Overseas Territories^22,57–59^. Using transplantation of seagrass cores or plugs, the most cost-effective restoration method ($29.75 ha^−1^) deployed in The Bahamas as of 2020^60^, restoring even half the 7,600 to 19,300 km^2^ of seagrass gained in our restoration scenario would cost over $11 million. More expensive methods of seagrass restoration can cost over $100,000 ha^−1^^61^. As these geographies are among the lowest contributors to global emissions yet are disproportionately vulnerable to the effects of climate change, SIDS could benefit from international assistance with these high costs, which could be supplied in part through carbon credits or other payment for ecosystem services mechanisms^25,62^.

We stress that blue carbon SOC accumulation is not a substitute for rapid global decarbonization in addressing the global climate crisis. SOC accumulation by mature seagrass is gradual over time. While the current average CARs are within ranges equivalent with the magnitude of GHG emissions of small countries with relatively low emissions such as The Bahamas, on a global scale, stabilizing the climate will require more than BCEs. Nature-based solutions as a whole have the potential to offset approximately one third of the emissions that need to be addressed to keep warming below 2 ^o^C^2^, and BCEs can contribute to ~ 3% of this climate target^63^.

Seagrass conservation and restoration provides benefits beyond carbon accumulation, such as water filtration to nearby coral reefs^17,64^. Seagrass meadows support higher biodiversity compared to the surrounding sandy habitat by providing a food source and refuge from predators^65–67^. Additionally, seagrass beds can counteract the effects of ocean acidification at a local scale with the right densities and abiotic conditions^68,69^, particularly for calcifiers and larval organisms that are more vulnerable to low pH^70^. Finally, this ecosystem holds cultural and spiritual value for numerous people^17^.

## Conclusions

Continued losses of seagrass at a rate of 1% per year in the Bahama Banks could result in losses of up to 200 Mt CO_2_ eq or gains of up to 800 Mt CO_2_ eq of stored SOC (Fig. 2). In contrast, meeting the restoration targets of the global biodiversity framework would, before the end of the century, result in additional SOC accumulation on par with the annual GHG emissions of The Bahamas, although additional carbon accumulation does not equate to avoided emissions. Timing of losses and restoration were large drivers of net SOC accumulation, with early restoration and delayed losses producing the greatest climate benefits over time, second only to maintaining intact seagrass. Models of carbon accumulation additionality for NCSs are critical to supporting development of robust NDCs and ensuring that natural ecosystems are adequately valued for the climate mitigation services they provide.

## Methods

### Site description

The Bahama Banks are shallow areas on average < 10 m deep with carbonate sediments located in the subtropical western Atlantic Ocean (25.026 ^o^N, 78.036 ^o^W), covering about 135,000 km^2^. They are composed of two major formations, Great Bahama Bank and Little Bahama Bank (Fig. 1), with a narrow “tongue” 1000 to 2000 m deep in between them. To the east, the bottom depth drops steeply to over 4000 m^71^. Mean monthly sea surface temperatures generally range from 23 to 31 ^o^C. Water temperatures are projected to warm 1.0 to 1.7 ^o^C by 2035 and 2.8 ^o^C by 2070, depending on greenhouse gas emission pathways^72^. With warm, clear water that allows high light penetration, the carbonate substrate can support seagrass growth in approximately 83% of the Bahama Banks, with up to 92,500 km^2^ being currently occupied with seagrass^26^. *Thalassia testudinum* is the dominant seagrass species in dense meadows, mixed with *Syringodium filiforme* in varying proportions^16,26^, while the sparser *Halodule wrightii* and *Halodule wrightii* are found higher-energy areas and cover a greater proportion of the Bahama Banks^26,31,73^.

### Seagrass extent scenarios

We modeled gains and losses in seagrass SOC in the Bahama Banks from 2020 to 2120 using the InVEST Coastal Blue Carbon Model version 3.14.0^74^. This model is frequently used to assess how accumulation in BCEs will change under different management scenarios^57,75,76^. We used two seagrass trajectories as scenarios: (1) the Business As Usual (BAU) scenario of 1% ± 0.5% (standard error; SE) loss in seagrass area per year, the median rate of loss for the tropical Atlantic region after 1900 (1.020% ± 0.497% per year, rounded to the nearest 0.1%)^20^ potentially due to nutrient pollution, coastal development, and other activities related to increased tourism; and (2) a restoration scenario meeting the targets of the Kunming-Montreal Framework, entailing stopping seagrass losses by 2030 and restoring seagrass extent to the inferred extent of 30 years prior based a reversal of the annual loss rate and suitable area, whichever was smaller^25^. For both of these scenarios, we used maps of a high estimate and a conservative estimate of initial seagrass extent at a 0.01-degree resolution, based on remote sensing and in-water camera surveys^26^. We assumed that these maps were representative of seagrass cover from 2020 to 2029. To model sensitivity of carbon accumulation estimates to rates of change, we added and subtracted the standard error to estimate the upper and lower bounds of the annual change rate: 0.5% and 1.5% (Table 1). These rates are similar to global average loss rates of seagrass^20^.

**Table 1:** Parameters used in the invest blue carbon model. Means ± 1 standard error (SE) are presented. “t CO_2_ eq” indicates metric tons of carbon dioxide equivalent. “SOC” indicates sediment organic carbon.

Parameter	Value	Source
Initial SOC stock (top 1 m of sediment)	251.4 ± 12.8 t CO_2_ eq ha^−1^	^26^
SOC accumulation rate	1.1 ± 0.3 t CO_2_ eq ha^−1^ yr^−1^	^16^
SOC loss from disturbance (classified as “low-impact disturbance” in InVEST)	40%	^8^
SOC accumulation rate of restored seagrass relative to mature seagrass, 0–10 years post-restoration)	75.0 ± 13.1% (maximum: 100%)	^33^^,^^84^
SOC accumulation rate of restored seagrass relative to mature seagrass, 10–20 years post-restoration)	96.5 ± 17.8% (maximum: 100%)	^33^^,^^84^
Half-life of SOC	3.8 years	^8^
Autochthonous SOC percentage	26.5%	^16^

For the restoration scenario, maps of seagrass extent 30 years prior were unavailable. To back-calculate seagrass area, we reversed the 1 ± 0.5% annual loss rate to a 1 ± 0.5% annual gain rate, equating to an inferred 34.8% higher seagrass area of 30 years prior. We assumed that all seagrass currently present was maintained through 2120 (Fig. 1). We constrained the maximum suitable restoration area to depths of ≤ 15 m regardless of whether expansion at this rate could exceed this area by 2120, as this is a practical constraint for restoration using SCUBA. Restored seagrass spreads beyond replanting zones through clonal growth and seed dispersal, and it can colonize greater depths^65,77^. Thus, the area we designated as eligible for restoration is an underestimate of the total area available for seagrass recolonization. When restorable area was combined with initial seagrass area, this produced a maximum suitable area of 100,579 km^2^. This amount was slightly less than the suitable area for seagrass growth in the Bahama Banks predicted by Gallagher et al.^26^. For each decade, we randomly selected 10% of restorable pixels to convert from non-seagrass to seagrass, with the maximum area restored in a decade being 2007 km^2^. When using the high estimate of initial seagrass area, less unvegetated area was available at depths ≤ 15 m compared to the conservative initial seagrass estimate, leading to less restoration area (Fig. 1).

The Coastal Blue Carbon Model uses a raster-based framework to track changes in land cover types and calculates the associated gains or losses in carbon. It assumes that land cover types remain the same until a new land cover map is provided, representing a specified date. We created decadal rasters of seagrass cover in R version 4.3.2^78^. At each timepoint, we summed the area (km^2^) of each pixel containing seagrass cover to estimate area change over time. For the BAU, this translated to a 10% loss per decade (Fig. 1). The pixels lost were randomly assigned. We assumed that seagrass loss would lead to sediment erosion and subsequent remineralization of carbon. Seagrass losses for the next decade were calculated based on the prior decade’s seagrass extent, with SOC loss being most severe in the first year following disturbance. Remaining seagrass continued to accumulate SOC, while SOC was lost from disturbed seagrass according to an exponential decay function:

1 $$\:{Emissions}_{t}=S*M*({0.5}^{\frac{t-\left(s+1\right)}{H}}-{0.5}^{\frac{t-s}{H}})$$

where *S* represents SOC stock in a given pixel, *M* represents the proportion of carbon lost during a disturbance of specific intensity, *t* represents the time as a year, *s* represents the year of disturbance, and *H* represents half-life of SOC^74^. Although half-life of SOC for seagrass slows over time^79^, this parameter has the greatest impact on emissions in the first few years following the disturbance, enabling use of a single value for half-life.

### Sediment organic carbon accumulation and loss

To parameterize the model’s organic carbon dynamics, we obtained baseline SOC stock and CARs from cores of *T. testudinum and S. filiforme* taken in November 2021 from Bahamian seagrass beds^16,26^. Core data was standardized to the top 1 m of sediment. We applied the same parameters to all seagrass species and vegetation densities. CARs were similar between the two seagrass species^16^. We were unable to find core data from *Halophila decipiens* or *Halodule wrightii* from The Bahamas, indicating a data gap, but comparisons to *T. testudinum* from other locations in the Caribbean showed that these species had similar ranges of SOC stocks and net ecosystem productivity despite having lower leaf biomass and net primary production^31,80–82^. We assumed that carbon stocks and CARs for Bahamas *Halophila decipiens* and *Halodule wrightii* were similar to those of the species with core data. However, because these species occupy higher energy environments with greater chances for sediment transport offsite^73^, these CARs may be considered optimistic.

Where seagrass cover was lost, we assumed that 40% of SOC in the top 1 m of sediment was remineralized to CO_2_ due to erosion and exposure to an oxic environment^8^, which was classified as a “low-impact disturbance” in InVEST^74^. This was the only disturbance type modeled. We did not model changes to OC stored in seagrass or epiphyte biomass, as the biomass typically has a short turnover time relative to sediment accumulation timescales^83^, and any seagrass litter that remained onsite that did not decompose would eventually be incorporated into the sediment.

Restored seagrass meadows typically take 1 to 2 decades for CARs to converge with that of mature meadows^33,84^, though the recovery of ecosystem area to its pre-disturbance state may take longer^85^. In the initial decades, restored meadows may have lower CARs. To calculate the proportionally reduced CAR of restored meadows, we divided the CARs of restored seagrass meadows by that of their undisturbed counterparts. We multiplied the baseline CAR by the mean proportions (± SE; Table 1) to determine CAR of restored meadows from 0 to 10 years and 10 to 20 years after restoration. We assumed that CARs remained constant on a decadal scale and that CARs resembled that of mature meadows after 20 years. Because NDCs and other carbon accounting methods are reported in terms of metric tons of carbon dioxide-equivalent, we converted the parameters for SOC stock and CAR to t CO_2_ eq ha^−1^. We converted the molecular weight of organic C to CO_2_ equivalent by multiplying by 3.67. We assumed that CARs for a given level of seagrass maturity would remain the same for the entire modeling period.

To calculate an upper and lower bound for SOC accumulation, we added and subtracted 0.5% from the seagrass’ 1% annual rates of change^20^. We represented the range of SOC stocks and CARs by using the mean ± SE for these parameters. For example, the upper bound of the restoration scenario had a 1.5% per year seagrass expansion rate and used the mean + 1 SE for SOC stock and CAR (Table 1).

Area differences between pixels selected for seagrass loss or restoration varied by a maximum of 0.15 km^2^ between the northern and southern end of the study area, owing to the latitudinal gradient in area contained within each 0.01-degree pixel. While predicting exact locations of seagrass loss or restoration was beyond the scope of this study, latitude-driven area variation in our simulation of random loss and restoration would induce a maximum of 15% error to our total net SOC accumulation estimates. The actual error induced is likely to be smaller than this because much of the seagrass was located in the middle latitudes of the study area, particularly in the restoration scenario (Fig. 1).

The Coastal Blue Carbon Model produces rasters of net SOC accumulation or loss per unit area. At the 50-year and 100-year timepoints (2070 and 2120, respectively), we multiplied the SOC raster values by the area of each cell to find total net SOC accumulation within a cell. We summed all cell values to find net SOC accumulation for the Bahama Banks in each scenario. We then subtracted the net SOC accumulation of BAU from the restoration scenario to estimate SOC additionality, or the difference from the status quo. We calculated autochthonous SOC accumulation and additionality by multiplying total SOC by the percentage of autochthonous carbon (Table 1). Stable isotope analyses of source material for carbon in seagrass sediments indicate 63.5% (69–78%) of SOC is derived from allochthonous sources, versus 26.5% (22–31%) derived from autochthonous material such as seagrass and epiphytes^16^.

## Data Availability

Seagrass organic carbon data is available on Figshare (https://doi.org/10.6084/m9.figshare.28890269). Questions can be directed to monica@beneaththewaves.org.
